# Supplementary material for: Long-term perturbation of the peripheral immune system months after SARS-CoV-2 infection
Source: BMC Med. 2022 Jan 14;20:26. doi: 10.1186/s12916-021-02228-6 (PMC8758383; doi:10.1186/s12916-021-02228-6)
Supplement: Supplementary file 1 — Additional file 1: Figure S1: Stability of anti-Spike and anti-RBD antibody titres over time. (A-E) Anti-Spike and (F-J) anti-RBD IgG, IgG1, IgG3, IgM and IgA titres plotted as a function of time. End point titres are reported as log10 area under the curve (AUC). The blue line represents the line of best fit from a linear regression analysis. The shaded areas represent the 95% confidence interval. The P value shown is from the linear regression. Red dashed lines represent the mean AUC + 2 SD in healthy controls for each isotype. [file 12916_2021_2228_MOESM1_ESM.pdf]

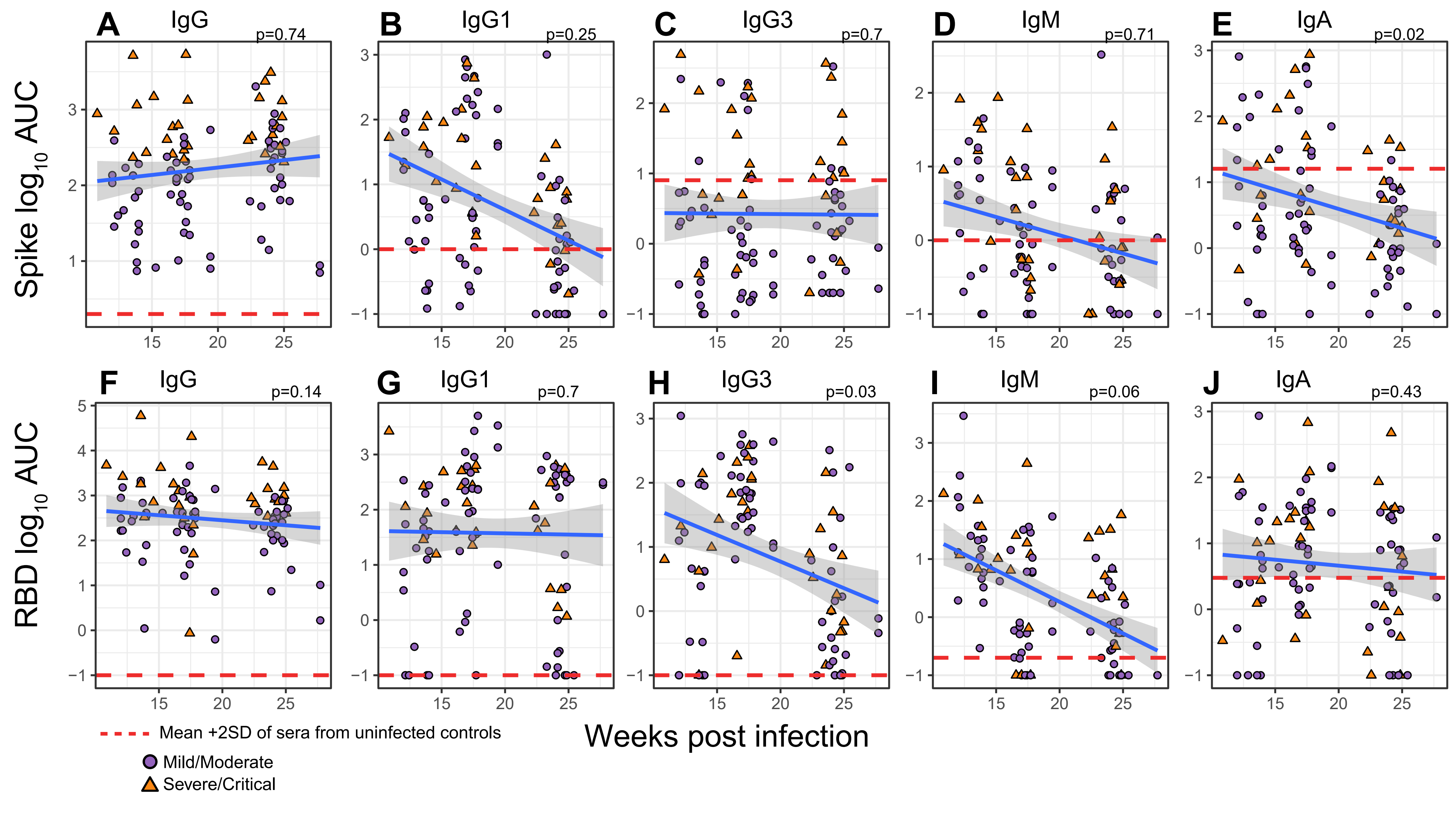

A
IgG
p=0.74
B
IgG1
p=0.25
C
IgG3
p=0.7
D
IgM
p=0.71
E
IgA
p=0.02
F
IgG
p=0.14
G
IgG1
p=0.7
H
IgG3
p=0.03
I
IgM
p=0.06
J
IgA
p=0.43
Spike log10 AUC
RBD log10 AUC
Weeks post infection
Mean +2SD of sera from uninfected controls
Mild/Moderate
Severe/Critical
