# Supplementary material for: Long-term perturbation of the peripheral immune system months after SARS-CoV-2 infection
Source: BMC Med. 2022 Jan 14;20:26. doi: 10.1186/s12916-021-02228-6 (PMC8758383; doi:10.1186/s12916-021-02228-6)
Supplement: Supplementary file 2 — Additional file 2: Figure S2: Expression of genes in two pathways identified as downregulated in COVID-19 convalescents and healthy controls. (A) Reactome pathway R-HSA-76002 “Platelet activation, signaling and aggregation” and (B) KEGG pathway hsa00190 “Oxidative phosphorylation”. Oxidative phosphorylation genes are sub-divided into nuclear and mitochondrially encoded, with the same x axis order of samples in each panel. Only differentially expressed genes (FDR < 0.05 and fold change > 1.25-fold) within each pathway are shown. (C) Serum CRP levels in samples collected from healthy controls (HC) and COVID-19 convalescent individuals at 12, 16 and 24 weeks post infection. [file 12916_2021_2228_MOESM2_ESM.pdf]

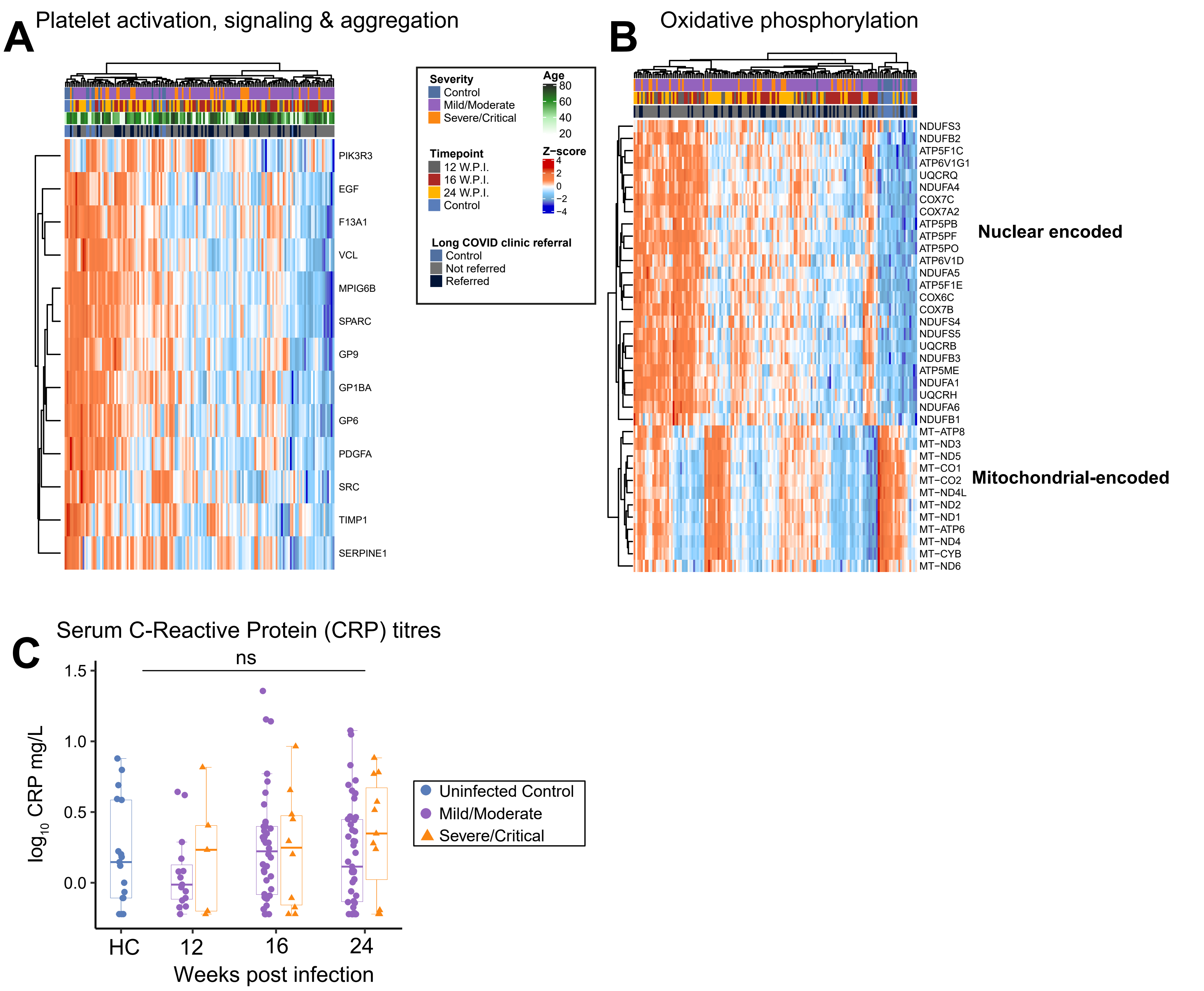

A
Platelet activation, signaling & aggregation
PIK3R3
EGF
F13A1
VCL
MPIG6B
SPARC
GP9
GP1BA
GP6
PDGFA
SRC
TIMP1
SERPINE1
Severity
Control
Mild/Moderate
Severe/Critical
Age
80
60
40
20
Timepoint
12 W.P.I.
16 W.P.I.
24 W.P.I.
Control
Z−score
4
2
0
−2
−4
Long COVID clinic referral
Control
Not referred
Referred
B
Oxidative phosphorylation
NDUFS3
NDUFB2
ATP5F1C
ATP6V1G1
UQCRQ
NDUFA4
COX7C
COX7A2
ATP5PB
ATP5PF
ATP5PO
ATP6V1D
NDUFA5
ATP5F1E
COX6C
COX7B
NDUFS4
NDUFS5
UQCRB
NDUFB3
ATP5ME
NDUFA1
UQCRH
NDUFA6
NDUFB1
MT−ATP8
MT−ND3
MT−ND5
MT−CO1
MT−CO2
MT−ND4L
MT−ND2
MT−ND1
MT−ATP6
MT−ND4
MT−CYB
MT−ND6
Nuclear encoded
Mitochondrial-encoded
C
Serum C-Reactive Protein (CRP) titres
ns
1.5
1.0
0.5
0.0
log10 CRP mg/L
HC
12
16
24
Weeks post infection
Uninfected Control
Mild/Moderate
Severe/Critical
