# Supplementary figures and images for: Long-term perturbation of the peripheral immune system months after SARS-CoV-2 infection
Source: BMC Med. 2022 Jan 14;20:26. doi: 10.1186/s12916-021-02228-6 (PMC8758383; doi:10.1186/s12916-021-02228-6)

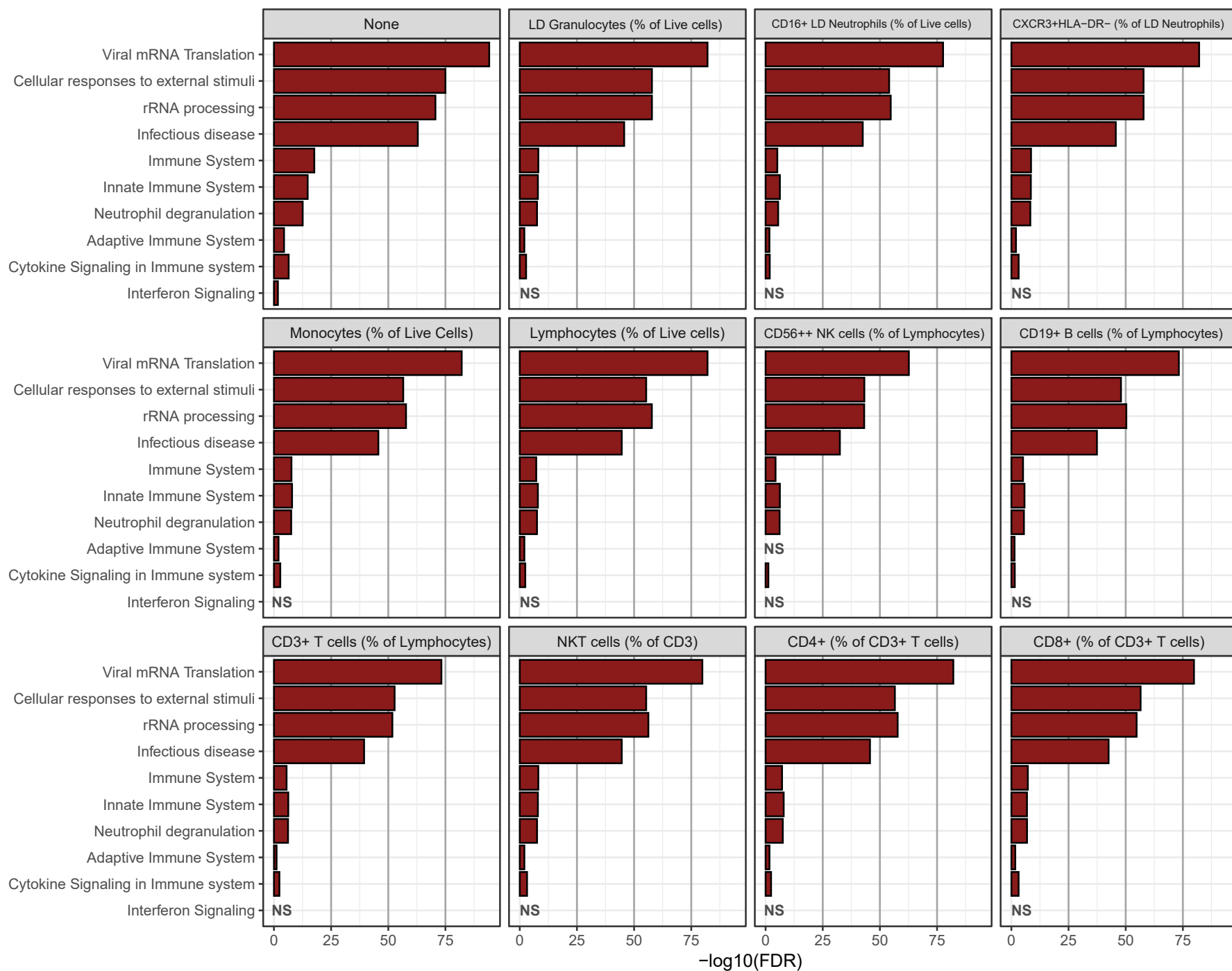

Supplement: Supplementary file 3 — Additional file 3: Figure S3: Adjusting for differences in immune cell populations. We repeated the differential expression analysis multiple times, each time adjusting for differences in the frequency of major immune cell populations among individuals. Each panel shows the enrichment of selected pathways (same as those shown in Fig. 4F) among genes identified as being significantly up-regulated in each analysis (FDR < 0.05 and fold change > 1.25-fold). None = no immune cell population adjustment. [file 12916_2021_2228_MOESM3_ESM.pdf]
