# Supplementary material for: Long-term perturbation of the peripheral immune system months after SARS-CoV-2 infection
Source: BMC Med. 2022 Jan 14;20:26. doi: 10.1186/s12916-021-02228-6 (PMC8758383; doi:10.1186/s12916-021-02228-6)
Supplement: Supplementary file 10 — Additional file 10. Symptom questionnaire sent to patients to assess presence of long COVID associated symptoms. [file 12916_2021_2228_MOESM10_ESM.pdf]

COVID-19 SA Study: Long COVID Questionnaire

Date	***Site Number***	***Study Number***	***Patient Initials***
☐☐/☐☐☐/☐☐☐☐	☐☐	☐☐☐	☐☐☐☐
(dd/mon/yyyy)			*E.g. SMITH, John SMJO*

1. Do you have any ongoing discomfort since you were discharged from hospital/released from isolation following COVID-19 infection? If yes, please specify

2. How would you describe your current health status?
□ Same as prior to COVID-19
□ Often feel fatigued, and tire more easily after activity now than prior to COVID-19
□ Better health status than prior to COVID-19

3. Have you experienced any of the following pain symptoms that appeared post COVID-19 and are persistent?
□ No pain
□ Headache
□ Myalgia (muscle ache)
□ Chest pain
□ Joint pain (if yes, please fill in the table below)
□ Any other if yes, please specify___________

Joints	*Hand*	*Foot*	*Wrist*	*Ankle*	*Jaw*	*Elbow*	*Shoulder*	*Neck*	*Hip*	*Knee*
Tenderness										
Swollen										
Numerical pain scale (0-10)										

0 **No pain** → **10** **Intolerable pain**

4. Do you have any of the following symptoms that are new following COVID-19 infection and still present?
□ None
□ Sore throat
□ Difficulty swallowing

5. Do you have any of the following symptoms that were not present before COVID-19 infection but are present now?
□ None
□ Low grade fever (37.3-38.0°C)
□ Palpitations
□ Dizziness
□ Nasal congestion
□ Skin rash

6. Are you more prone to experience the following symptoms since recovering from COVID-19 infection?
□ None
□ Diarrhoea
□ Nausea
□Vomiting

7. How do you feel about your sense of smell now as compared to prior to COVID-19 infection?
□ Same as before
□ Worse than before
□ Better than before
□ Total loss

8. How do you feel about your sense of taste now as compared to prior to COVID-19 infection?
□ Same as before
□ Worse than before
□ Better than before
□ Total loss

9. How do you feel about your appetite now as compared to prior to COVID-19 infection?
□ Same as before
□ Worse than before
□ Better than before

10. What do you think about your sleeping now as compared to prior to COVID-19?
□ Same as before
□ Worse than before
□ Better than before

11. How do you feel about your muscle strength now as compared with the status prior to COVID-19?
□ Same as before
□ Worse than before
□ Better than before

12. How do you experience hair loss now as compared with prior to COVID-19?
□ No hair loss before or after COVID-19
□ Hair loss is same as before
□ Lose more hair now than before
□ Lose less hair now than before

13. How do you feel about your memory and concentration now as compared with prior to COVID-19?
□ Same as before
□ Worse than before
□ Better than before

Assess your health status TODAY and BEFORE COVID-19

Under each heading please tick the ONE box that best describes your health TODAY and IMMEDIATELY PRIOR TO BEING DIAGNOSED WITH COVID-19.

MOBILITY

TODAY

□ I have no problems in walking about
□ I have slight problems in walking about
□ I have moderate problems in walking about
□ I have severe problems in walking about
□ I am unable to walk about

BEFORE COVID-19

□ I had no problems in walking about
□ I had slight problems in walking about
□ I had moderate problems in walking about
□ I had severe problems in walking about
□ I was unable to walk about

SELF-CARE

TODAY

□ I have no problems with washing or dressing myself
□ I have slight problems with washing or dressing myself
□ I have moderate problems with washing or dressing myself
□ I have severe problems with washing or dressing myself
□ I am unable to wash or dress myself

BEFORE COVID-19

□ I had no problems with washing or dressing myself
□ I had slight problems with washing or dressing myself
□ I had moderate problems with washing or dressing myself
□ I had severe problems with washing or dressing myself
□ I was unable to wash or dress myself

Date	Site Number	Study Number	Patient Initials
□□/□□□/□□□□	□□	□□□	□□□□
(dd/mon/yyyy)			E.g. SMITH, John SMJO

USUAL ACTIVITIES *(e.g. work, study, housework, family or leisure activities)*

TODAY

□ I have no problems doing my usual activities
□ I have slight problems doing my usual activities
□ I have moderate problems doing my usual activities
□ I have severe problems doing my usual activities
□ I am unable to do my usual activities

BEFORE COVID-19

□ I had no problems doing my usual activities
□ I had slight problems doing my usual activities
□ I had moderate problems doing my usual activities
□ I had severe problems doing my usual activities
□ I was unable to do my usual activities

PAIN / DISCOMFORT

TODAY

□ I have no pain or discomfort
□ I have slight pain or discomfort
□ I have moderate pain or discomfort
□ I have severe pain or discomfort
□ I have extreme pain or discomfort

BEFORE COVID-19

□ I had no pain or discomfort
□ I had slight pain or discomfort
□ I had moderate pain or discomfort
□ I had severe pain or discomfort
□ I had extreme pain or discomfort

ANXIETY / DEPRESSION

TODAY

□ I am not anxious or depressed
□ I am slightly anxious or depressed
□ I am moderately anxious or depressed
□ I am severely anxious or depressed
□ I am extremely anxious or depressed

BEFORE COVID-19

□ I was not anxious or depressed
□ I was slightly anxious or depressed
□ I was moderately anxious or depressed
□ I was severely anxious or depressed
□ I was extremely anxious or depressed

Date	***Site Number***	***Study Number***	***Patient Initials***
☐☐/☐☐☐/☐☐☐☐	☐☐	☐☐☐	☐☐☐☐
(dd/mon/yyyy)			*E.g. SMITH, John SMJO*

- We would like to know how good or bad your health is **TODAY**.
- This scale is numbered from 0 to 100.
- 100 means the best health you can imagine.
  0 means the worst health you can imagine.
- Mark an X on the scale to indicate how your health is **TODAY**.
- Now, please write the number you marked on the scale in the box below

The best health you can imagine

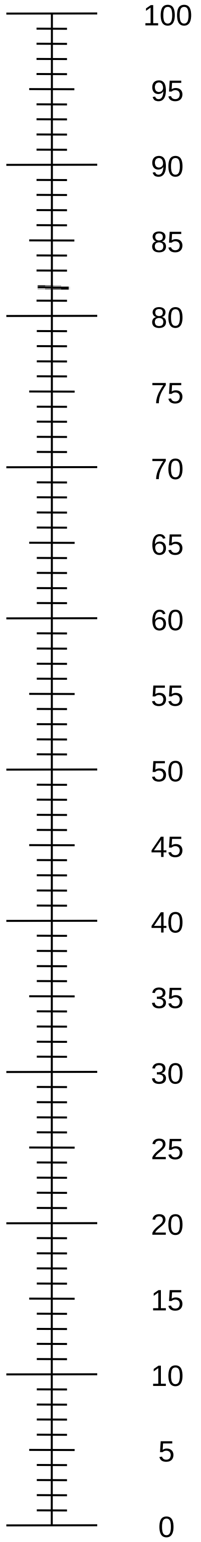

The worst health you can imagine

Date	*Site Number*	*Study Number*	*Patient Initials*
☐☐/☐☐☐/☐☐☐☐	☐☐	☐☐☐	☐☐☐☐
(dd/mon/yyyy)			*E.g. SMITH, John SMJO*

The best health you can imagine

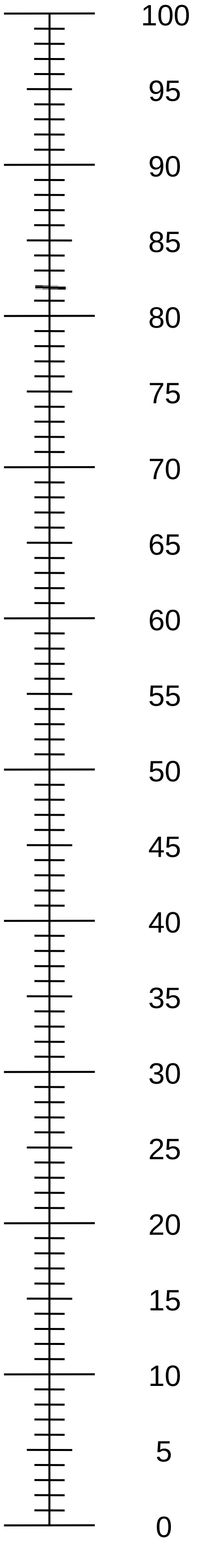

The worst health you can imagine

- We would like to know how good or bad your health **<u>WAS IMMEDIATELY PRIOR TO BEING DIAGNOSED WITH COVID-19.</u>**
- This scale is numbered from 0 to 100.
- 100 means the <u>best</u> health you can imagine.
  0 means the <u>worst</u> health you can imagine.
- Mark an X on the scale to indicate how your health is **<u>WAS IMMEDIATELY PRIOR TO BEING DIAGNOSED WITH COVID-19</u>**.
- Now, please write the number you marked on the scale in the box below

Date	***Site Number***	***Study Number***	***Patient Initials***
☐☐/☐☐☐/☐☐☐☐	☐☐	☐☐☐	☐☐☐☐
(dd/mon/yyyy)			*E.g. SMITH, John SMJO*

Chose the phrase that best describes your breathing before and after COVID-19 according to the following table

Box 3: **Modified Medical Research Council (mMRC) Dyspnoea Scale for grading the severity of breathlessness during daily activities**

Grade	Symptom complex
0	I only get breathless with strenuous exercise
1	I get short of breath when hurrying on level ground or walking up a slight hill
2	On level ground, I walk slower than people of the same age because of breathlessness, or I have to stop for breath when walking at my own pace on the level
3	I stop for breath after walking about 100 metres or after a few minutes on level ground
4	I am too breathless to leave the house or I am breathless when dressing or undressing

Before COVID-19 grade: ___________________

After COVID-19 grade: ___________________
